# Healthy hearts: a cross-sectional study of clinical cardiovascular disease risk factors in Northern Colorado school children (1992–2013)

**DOI:** 10.1186/s40608-015-0078-9

**Published:** 2015-12-09

**Authors:** Tracy L. Nelson, NaNet Puccetti, Gary J. Luckasen

**Affiliations:** Department of Health and Exercise Science, Colorado State University, 220 Moby Complex, Fort Collins, Colorado 80523 USA; Healthy Hearts Program Supervisor, University of Colorado Health Research-Northern Region, 2500 Rocky Mountain Avenue, South Medical Office Building, Suite #360, Loveland, Colorado 80538 USA; Medical Director of University of Colorado Health Research-Northern Region, 2500 Rocky Mountain Ave., SMOB -Suite 360, Loveland, Colorado 80538 USA

**Keywords:** Cardiovascular risk factors, Children, Overweight and obese, Family influence

## Abstract

**Background:**

Despite significant declines in cardiovascular disease (CVD), it remains the number one cause of death in the United States. Determining factors that may be associated with CVD risk at a young age may allow us to better prevent CVD deaths in the future. The purpose of this paper is to determine the prevalence of CVD risk factors among 4^th^ grade children who participated in a community-wide CVD education program; as well as the association of these risk factors with weight status and the prevalence of CVD risk factors among family members.

**Methods:**

The Poudre Valley Health Systems, Healthy Hearts Club has provided a cardiovascular screening program (1992–2013) to identify risk factors among students in six Northern Colorado school districts. There were 9,694 children (mean age, 10.3 years, 50 % female) included. Data were collected cross-sectionally with objective measures of total and high-density lipoprotein cholesterol (HDL-C), blood pressure and body mass index (BMI). Surveys were filled out by the parent and/or legal guardian and included questions about risk factors among family members. Means and frequencies were compared using SPSS software version 22 (IBM, Inc.).

**Results:**

There were a significant number of children with elevated risk factors, including 35 % with total cholesterol ≥ 170 mg/dl, 22 % with HDL-C < 40 mg/dl, 13 % with Non-HDL-C ≥ 145 mg/dL, 6 % and 7 % with systolic and diastolic blood pressure ≥ 120 mmHg, and ≥ 80 mmHg respectively, and 21 % with BMI ≥ 85 % for age and sex. All the risk factors increased significantly when comparing normal weight to overweight and obese children. Further, among children with zero risk factors, 32.2 % reported a family member (other than the child) being overweight, while 56.5 % reported such among those children with five or six risk factors.

**Conclusions:**

Overall, the prevalence of CVD risk factors in these children is similar to national levels and these factors are meaningfully associated with overweight and obesity, both within the child as well as within the family. This data suggests CVD risk factor reduction and prevention must focus on overweight and obesity and not be done in isolation of the family.

## Background

Despite significant declines in cardiovascular disease (CVD) since the 1950’s, CVD is still the number one cause of death in the United States. Many of the risk factors for CVD, including risk behaviors, start early and carry over to adulthood (see Lloyd-Jones et al. [1] review); and unfortunately their prevalence among youth is substantial. Recent National Health and Nutrition Examination Survey (NHANES) data shows that 8.1 % of youth 6–19 years. have total cholesterol ≥ 200 mg/dL, and 14.8 % have low high-density lipoprotein cholesterol (HDL-C) < 40 mg/dL; further, among males and females ages 8–17 years., 19.2 % and 12.6 % respectively, were found to have elevated blood pressure [2, 3], while ~34 % of children and adolescents were above the 85^th^ percentile of body mass index (BMI) for age and sex (e.g. overweight or obese) [4].

Primordial prevention of CVD risk factors in children, that is preventing their development in the first place, as well as primary prevention, addressing risk factors before they lead to disease, are primary goals of the American Heart Association (AHA) and the Expert Panel on Integrated Guidelines for Cardiovascular Health and Risk Reduction in Children and Adolescents [1, 5]. By considering exposures that may be associated with CVD risk factors in children we may gain a better grasp on how to effectively prevent such factors from occurring in the first place.

The purpose of this paper is to determine the prevalence of CVD risk factors among 4^th^ grade children who participated in a community-wide CVD education program in Northern Colorado from 1992–2013; and to consider exposures that may be associated with these risk factors, particularly weight status and the prevalence of CVD risk factors among family members.

## Methods

The Poudre Valley Health System (PVHS), Healthy Hearts Club has provided a successful cardiovascular screening program for the past ~21 years (1992–2013) to identify risk factors among students in six Northern Colorado school districts (a primarily white population ~90 %). Schools were selected based on willingness to participate. Each school who participated did so a maximum of one time per school year; schools who participated varied from year to year throughout the six school districts. Data were collected cross-sectionally every year, except 1997 and 1999, beginning in 1992 through 2013. Objective measures of non-fasting total and high-density lipoprotein cholesterol (HDL-C), blood pressure and body mass index were calculated. Surveys were filled out by the parent and/or legal guardian and included questions about diet and physical activity of the child as well as CVD risk factors among family members. Parental/guardian consent was obtained for all participants by having the parent/guardian sign a consent form at the time they filled out the health history questionnaires. The schools involved sent these consent forms home with the children. The current analysis included anonymous secondary data of information collected as part of this community outreach program; therefore, this study was determined not to meet the federal definition of human subject’s research by the Institutional Review Board at Colorado State University.

### Total cholesterol and HDL-C

Cholesterol was determined using venipuncture to obtain samples through 2000 and then the Cholestech LDX Finger Stick Test was used beginning in 2001. Collection of samples was done with the Cholestech LDX capillary tubes. Both total and HDL-C cassettes were used for analysis. Cholesterol values were non-fasting and one sample was obtained for each child. Acceptable total cholesterol was defined as < 170 mg/dL; borderline total cholesterol ≥ 170–199 mg/dL and high total cholesterol ≥ 200 mg/dL. Low HDL-C was defined as < 40 mg/dL and high non-HDL-C as ≥ 145 mg/dL [1, 5].

### Body mass index

Height was measured using a Secastadiometer and weight was measured using a DETECTO electronic scale. Height and weight were measured one time for each child. BMI percentile was calculated based on height, weight, age and sex using the most recent Centers for Disease Control (CDC) BMI calculator with overweight defined as BMI ≥ 85 and < 95 % and obesity defined as BMI ≥ 95 % [6].

### Blood pressure

Blood pressure was measured with a sphygomomanometer by WelchAllynfitted with appropriately sized cuffs. Blood pressure was taken two times for each child and the values were averaged. Elevated blood pressure was defined as either systolic blood pressure (SBP) ≥ 120 mmHg and/or diastolic blood pressure (DBP) ≥ 80 mmHg [1].

### Health history questionnaire

This survey was filled out by parents or legal guardians and contained 11 multilayered questions about nutrition, lifestyle, and family history.

#### Statistical methods

**Fig. 1:**
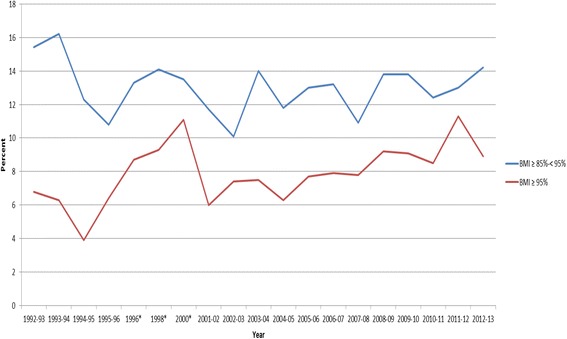
Percentage of Children Overweight (≥ 85 % –< 95 %) or Obese ≥ 95 %) School Years 1992–2013. Legend: BMI – body mass index; *No data 1997 or 1999

**Fig. 2:**
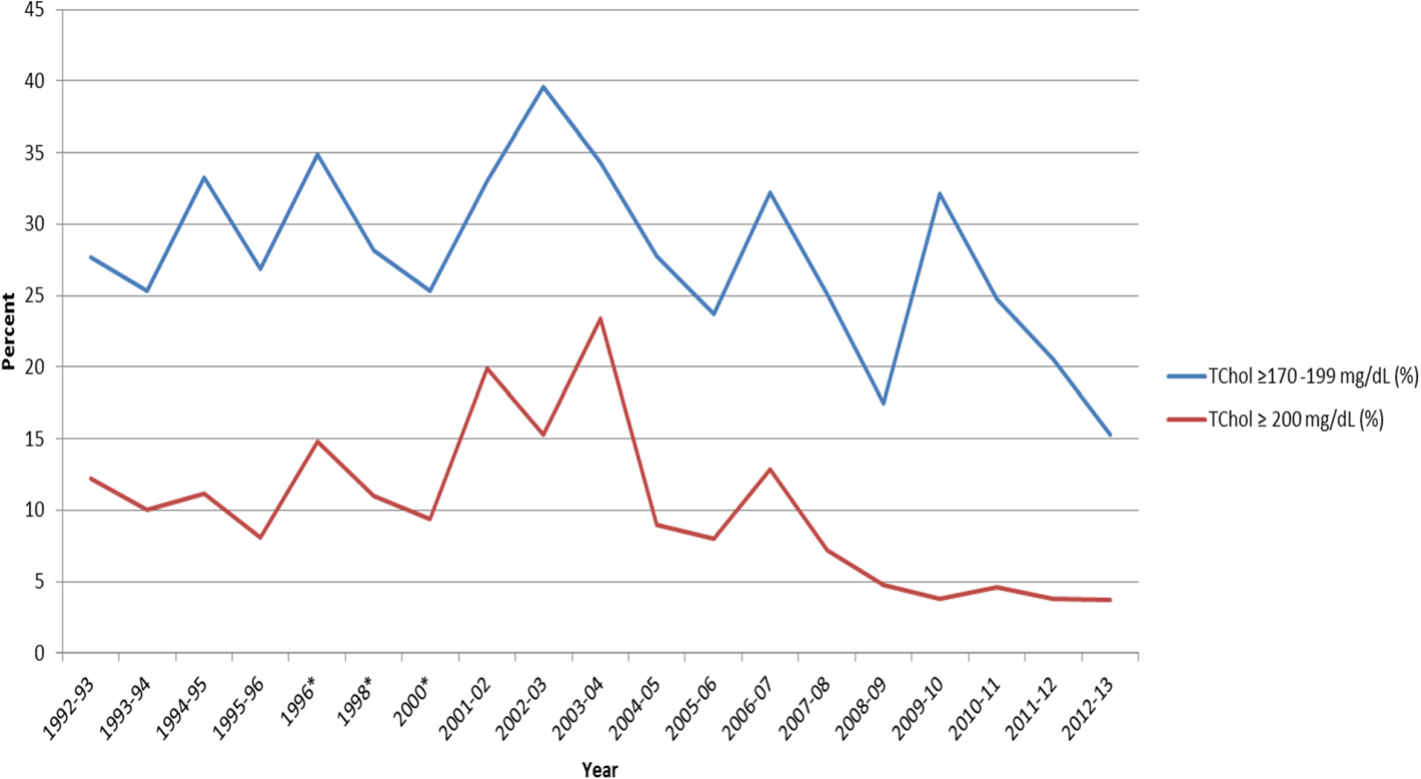
Percentage of Children with Borderline ≥ 170–199 mg/dL) or High Total Cholesterol ≥ 200 mg/dL) for School Years 1992–2013. Legend: TChol - Total Cholesterol; *No data 1997 or 1999

**Fig. 3:**
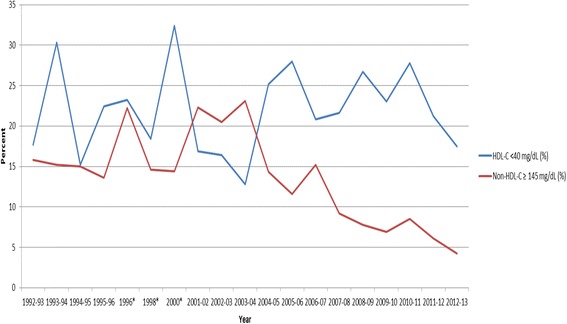
Percentage of Children with low HDL-C (<40 mg/dL) or Elevated Non-HDL-C ≥145 mg/dL) for School Years 1992–2013. Legend: HDL-C – High-density lipoprotein cholesterol; *No data 1997 or 1999

**Fig. 4:**
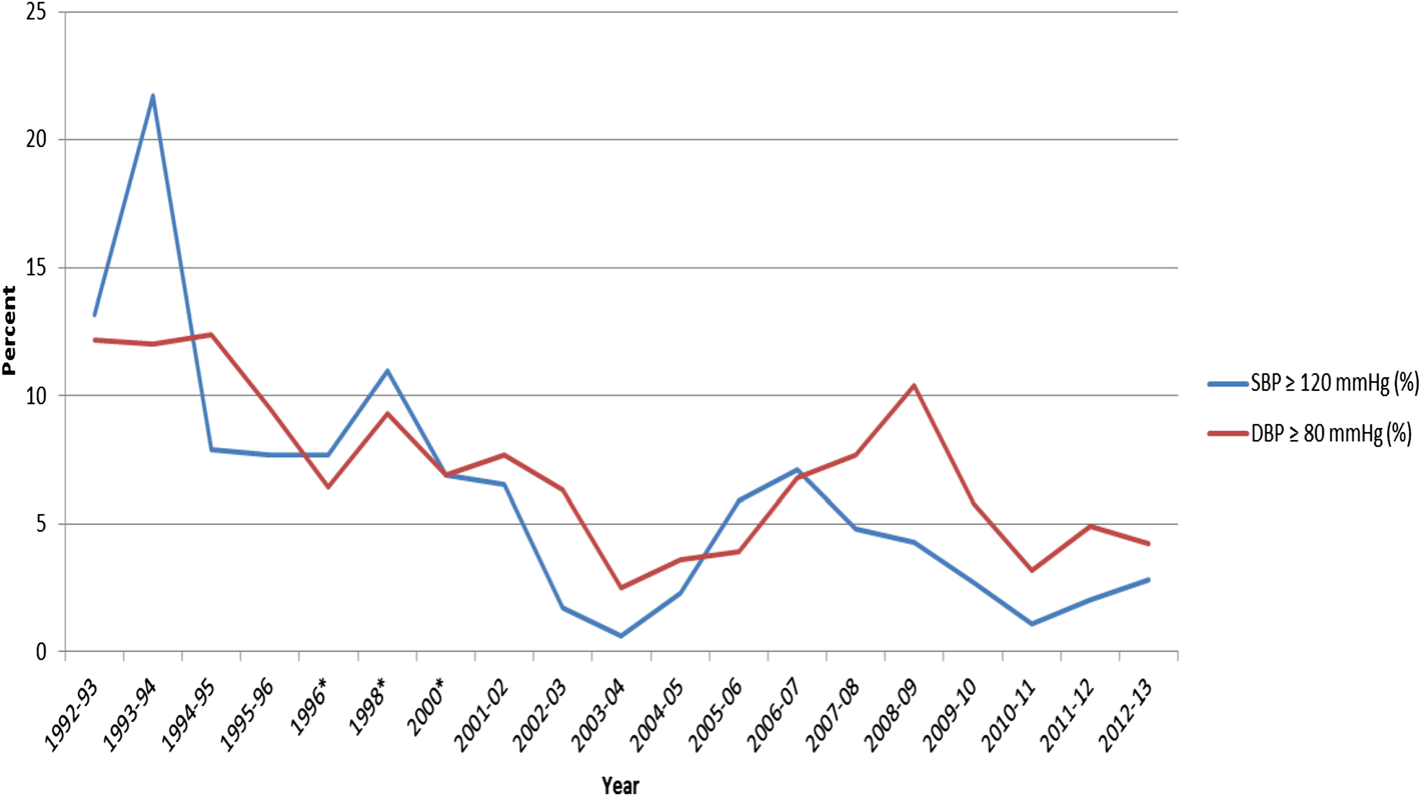
Percentage of Children with Elevated Systolic Blood Pressure (≥ 120 mmHg) or Diastolic Blood Pressure ≥ 80 mmHg) for School Years 1992–2013. Legend: SBP – systolic blood pressure; DBP – diastolic blood pressure; *No data 1997 or 1999

**Fig. 5:**
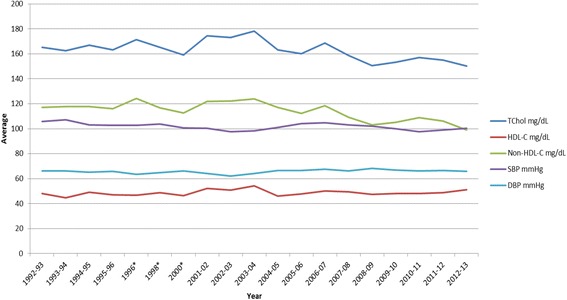
Average levels of Total Cholesterol, HDL-C, Non-HDL-C, SBP, DBP by School Years 1992–2013. Legend: TChol – total cholesterol; HDL-C – high-density lipoprotein cholesterol; SBP -systolic blood pressure; DBP – diastolic blood pressure; *No data 1997 or 1999

Mean levels of CVD risk factors were calculated for the entire sample by gender. An independent t-test was used to compare means by gender (Table 1). Frequencies of elevated levels of CVD risk factors were compared by gender using the Kruskall-Wallis test for nonparametric data (Table 2). Means and frequencies were also calculated for these risk factors by school year beginning in 1992–93 and ending with 2012–2013 (Figs. 1, 2, 3, 4 and 5). Data were not collected in 1997 or 1999 due to lack of funding. Means and frequencies for the CVD risk factors were also compared by normal, overweight and obese status, using a Kruskall-Wallis test (Table 3). Frequencies were calculated for the self-reported risk factors among family members (Table 4), where family members were asked yes or no to the following question “does anyone in your family (Mom, Dad, brothers, sisters) have: high blood pressure, high cholesterol, an overweight problem or diabetes. Family members were also asked: “does anyone in your family smoke? If yes, in the home or car?” as well as “how would you describe your health in general, as excellent, very good, good, fair or poor?” This survey was filled out by the parent or legal guardian. These self-reported risk factors among family members were then compared to the frequency of objectively measured risk factors in children, where 1 point was given for each of the following: total cholesterol ≥ 170; HDL-C < 40 mg/dL; Non-HDL-C ≥ 145 mg/dL; BMI percentile ≥ 85 %; SBP ≥ 120 mmHg; or DBP ≥ 85 mmHg, for a total score ranging from 0–6 (note: scores of 5 and 6 were combined due to low numbers). The Kruskall-Wallis test was used to test for significant differences in self-reported risk factors in family members by the risk factor scores (e.g. 0–6) in children. All statistical analyses were calculated using SPSS software version 22 (IBM, Inc.)

**Table 1:** Cardiovascular risk factors among male and female children participating in the healthy hearts club 1992–2013, values are expressed as mean (S.D.)

	Mean	S.D.	Mean	S.D.	*P*-value
	Males (*N* = 4851)		Females (*N* = 4843)		
Age (years)	10.33	0.58	10.28	0.63	<0.001
BMI (kg/m^2^)	17.97	3.36	17.90	3.22	NS
Total Cholesterol (mg/dL)	161.73	28.89	161.72	28.26	NS
HDL-C (mg/dL)	49.07	12.31	48.23	11.95	<0.001
Non-HDL-C (mg/dL)	112.66	29.35	113.48	28.25	NS
Systolic Blood Pressure (mmHg)	102.03	10.06	101.45	9.96	<0.05
Diastolic Blood Pressure (mmHg)	66.00	8.41	65.58	8.16	<0.05

*S.D*. standard deviation, *BMI* body mass index, *HDL*-*C* high-density lipoprotein cholesterol, *NS* not significant at *p* < 0.05

**Table 2:** Percentage of male and female children participating in the healthy hearts club 1992–2013 with elevated levels of CVD risk factors, values are expressed as percentages

	Percentage	Percentage	*P*-value
	Males (*N* = 4851)	Females (*N* = 4843)	
BMI ≥ 85 % – < 95 % (%)	13.7	12.3	<0.001
BMI ≥ 95 % (%)	9.3	6.9	<0.001
Total Cholesterol ≥170–199 mg/dL (%)	26.5	26.0	NS
Total Cholesterol ≥200 mg/dL (%)	9.4	9.4	NS
HDL-C < 40 mg/dL (%)	21.2	23.2	<0.05
Non-HDL-C ≥ 145 mg/dL (%)	13	12.9	NS
Systolic Blood Pressure ≥ 120 mmHg (%)	6.2	5.8	NS
Diastolic Blood Pressure ≥ 80 mmHg (%)	7.6	6.8	NS

*BMI* body mass index, *HDL*-*C* high-density lipoprotein cholesterol, *NS* not significant *p* < 0.05

**Table 3:** Cardiovascular risk factors by weight status among children participating in the healthy hearts club (Means/Frequencies)

	Normal	Overweight	Obese	*P*-value
	*N* = 7645	*N* = 1263	*N* = 786	
TChol mg/dL (SD)	160.2 (27.8)	166.2 (30.5)	169.5 (30.6)	<0.001
TChol ≥ 170–199 mg/dL (%)	25.5	28.3	30.8	<0.001
TChol ≥ 200 mg/dL (%)	8.1	13.4	15.1	<0.001
HDL-C mg/dL (SD)	50.1 (12.0)	44.8 (11.6)	40.9 (9.9)	<0.001
HDL-C < 40 mg/dL (%)	17.8	32.9	47.5	<0.001
Non-HDL-C mg/dL (SD)	110.1 (27.5)	121.3 (30.5)	128.6 (31.0)	<0.001
Non-HDL-C ≥ 145 mg/dL (%)	10.4	19.6	27.0	<0.001
SBP mmHg (SD)	100.4 (9.5)	105.0 (9.9)	109.5 (10.0)	<0.001
SBP ≥120 mmHg (%)	3.9	10.%	19.0	<0.001
DBP mmHg (SD)	65.0 (8.0)	67.6 (8.2)	71.0 (8.5)	<0.001
DBP ≥ 85 mmHg (%)	5.4	9.9	19.8	<0.001

*BMI* body mass index, *DBP* diastolic blood pressure, *HDL*-*C* high-density lipoprotein cholesterol, *SBP* systolic blood pressure, *TChol* Total Cholesterol, *SD* standard deviation

**Table 4:** Self-reported CVD risk factors in family members and number of measured child risk factors in healthy hearts club participants 1992–2013 (*N* = 9653)^b^. Number of Risk Factors in Children ^a^

Family member self-report risk factors	0	1	2	3	4	5-6	*P*-value
	*N* = 3706	*N* = 3262	*N* = 1648	*N* = 653	*N* = 301	*N* = 83	
Overweight (%)	32.2	33.4	41.1	51.4	59.4	56.5	<0.001
Diabetes (%)	15	13.2	14.6	19.4	18.3	10.0	NS
High Cholesterol (%)	24.2	25.5	26.1	27.6	27.2	23.1	<0.05
High Blood Pressure (%)	22.3	21.2	24.0	26.6	22.6	15.4	NS
Describe Health (% Fair or Poor)	32.3	28.8	22.8	26.9	33.3	50.0	NS
Smoke (%)	14.3	15.8	17.2	15.1	20.2	17.2	NS
Smoke Inside (%)	7.8	8.9	11.6	10.6	15.7	6.3	<0.05

*NS* not significant less than *p* < 0.05

^a^Possible risk factors in children: systolic blood pressure ≥ 120; diastolic blood pressure ≥ 80; total cholesterol ≥170; high-density lipoprotein cholesterol < 40; non- high-density lipoprotein cholesterol ≥ 145 mg/dL; body mass index ≥ 85 %

^b^41 children were missing one of the measured risk factors

## Results

Table 1 shows demographic data and the CVD risk factor averages across the 21 year period (1992–2013) by gender. Table 2 shows the frequency of elevated CVD risk factors by gender, where we found ~23 % of males and ~19 % of females to be overweight or obese and ~35 % of both genders to have borderline to high total cholesterol. Figures 1, 2, 3 and 4 show the percentage of children with elevated CVD risk factors by year between 1992 and 2013, while Fig. 5 shows the mean levels of the CVD risk factors by year. Most of the risk factors trended toward more favorable values (except overweight and obesity) with year-to-year fluctuations. Differences in these risk factors by overweight and obesity status are shown in Table 3, with all risk factors being significantly more unfavorable among overweight and obese children when compared to normal weight children. For example, 15.1 % of those who were obese had total cholesterol ≥ 200 mg/dL which was significantly more children than the 8.1 % with high total cholesterol levels among the normal weight children. There were no significant differences by gender for these associations (data not shown). Self-reported risk factors among parents/family members were also considered in relation to children’s objective risk factors (Table 4); we found that parents/family self-reported overweight showed the greatest difference in relation to the children’s CVD risk factors. For example, of those children with zero risk factors 32.2 % reported a family member (other than the child) being overweight while 56.5 % reported such among those children with five to six risk factors. The differences for the other self-reported parent/family risk factors were not nearly as pronounced.

## Discussion

The levels of cardiovascular disease risk factors among these Northern Colorado children are similar to those nationally, with many of the risk factors trending downward over this 21 year period. Unfortunately, these risk factors are highly associated with overweight and obesity which are not trending downward. Given these children are on average only 10 years old, finding clinically meaningful differences in CVD risk factors by weight status is important when considering future risk among these children [1, 5]. It is also important when we think about primordial and primary prevention; although our data are cross-sectional, our results suggest that focusing on reducing and eliminating overweight and obesity among children by way of focusing on the family may be the best type of intervention.

As noted previously, when comparing the CVD risk factor levels in this population to national data, we found similar values with average total cholesterol ~ 160 mg/dL nationally among children 6–19 years, and ~162 mg/dl among our population; [2] average national SBP and DBP are ~106 mmHg and ~58 mmHg respectively and ~102 mmHg and 66 mmHg among our population [3]; average overweight/obesity is ~34 % nationally among 6–19 years olds [6] which is quite a bit higher than our study where ~ 21 % were overweight/obese. We found similar values in males and females for these variables; similar to the other studies [2, 3, 7].

When considering the trends in risk factors over time, we again found similar results to those nationally, with most risk factors trending toward more favorable values. For example, the National Health and Nutrition Examination Survey (NHANES, 1988–2010) has shown serum lipids among U.S. youth 6–19 years to favorably decline, and HDL-C to increase, similar to our data. Blood pressure showed minor increases during this time period nationally in contrast to a downward trend in our data. Similar to national data, we did not see trends toward decreasing percentages of overweight/obese children [2, 3, 7].

We were also interested in exposures that may be associated with these CVD risk factors including overweight and obesity as well as the prevalence of risk factors among family members. We found overweight and obese children to have significantly higher levels of CVD risk factors than normal weight children. Specifically, obese children had a ~5 % higher total cholesterol, ~18 % lower HDL-C level, ~14 % higher non-HDL-C level, and ~8 % higher SBP and DBP than normal weight children. Such findings are consistent with the literature where Cottrell et al. found similar percent differences in risk by BMI status among 80,000, 10–12 year olds from the Coronary Artery Risk Detection in Appalachian Communities (CARDIAC) project [8]. Interestingly, data from the Bogalusa Heart study showed that being overweight in childhood was associated with adult carotid intima-media thickness, suggesting that long-term exposure to such CVD risk factors can have implications for future coronary artery disease [9]. Our data along with these studies suggest that aiming our efforts at both preventing and treating obesity in children may be one of our most effective means of preventing cardiovascular disease later in life.

We also considered the association of CVD risk factors among family members with risk factors among the children. We found that among children with five to six CVD risk factors (as described in the methods), 56.5 % of the parents/family members reported being overweight compared to children with 0 risk factors, where ~32.2 % reported being overweight. This is not surprising given that parent’s guide the health behaviors and practices of the children [10, 11] and that overweight and obesity were highly correlated with CVD risk factors in our population. When we only considered families reporting an overweight family member, we found 32 % of the children in this group were either overweight or obese; while only 14 % of children were overweight or obese among those families who did not report an overweight family member (data not shown). These findings support the strong association between family CVD risk factors, particularly being overweight and children’s risk factors. Further, these results corroborate a study of 7-year old children from Oulu, Finland (*n* = 855) where they found childhood obesity was most predicted by mother’s obesity followed by low physical activity and skipping breakfast [12]. Overall, family lifestyles may have a major influence on the future habits of children, including diet, physical activity, and smoking that eventually influence clinical outcomes including hypertension, diabetes mellitus, obesity, and dyslipidemia [1, 10]. If we can educate parents/families about the strong associations between weight status and CVD risk factors; focusing more on the “implications” of weight status, we may find more success in our interventions aimed at preventing and treating overweight and obesity [13]. In non-published data we found parents reported being more willing to try and change unhealthy lifestyle habits if they knew it was negatively impacting their child’s health; further, it has been shown that family interventions succeed over individual level interventions. A study by Epstein et al. found obese children assigned with an obese parent to diet, exercise and behavior management training showed reduced obesity compared to those children not assigned with a parent [14].

This study has some limitations. Cholesterol levels were determined in the non-fasting state; however, recent evidence supports the use of non-fasting lipid levels. Numerous studies, including a community-based population study, have found fasting time to be minimally associated with lipid levels [15, 16]; we therefore do not believe the use of non-fasting samples altered our data in any meaningful way. This data was collected cross-sectionally over a 21 year time period, therefore, we could not report on individual changes in risk factors over time and we could not determine if overweight or obesity preceded CVD risk levels in children, only that they are associated. Similarly, we do not know that family risk factors preceded the child’s risk factors, only that they are associated. The family risk factor profiles were all self-reported and so there could be some misclassification bias; however this was likely random and would therefore not be expected to influence the associations with the children’s CVD risk factors. This study was conducted in a primarily white population and therefore caution is advised in generalizing these results to other populations. Strengths of this paper include the objective measures of CVD risk markers collected and analyzed over this 21 year period in conjunction with the data obtained on family members.

## Conclusions

Levels of cardiovascular disease risk factors among these Northern Colorado children are similar to those nationally, with many of the risk factors trending downward. Unfortunately, these risk factors are highly associated with overweight and obesity which are stable or increasing; suggesting future cohorts may not see this downward trend in CVD risk factors. Although this data is cross-sectional, it suggests that to prevent risk factors at the primordial or primary level we must focus on overweight and obesity among children, in the context of reducing risk for CVD. Further these interventions should not isolate the child, but be inclusive of the family, given the strong associations found between children’s CVD risk factor levels and the prevalence of overweight among families.

## Abbreviations

CVD: Cardiovascular disease
NHANES: National Health and Nutrition Examination Survey
BMI: Body mass index
HDL-C: High-density lipoprotein cholesterol
AHA: American Heart Association
PVHS: Poudre Valley Health System
SBP: Systolic blood pressure
DBP: Diastolic blood pressure
CARDIAC: Coronary Artery Risk Detection in Appalachian Communities
